# Comparison of Protein and Carbohydrate Consumption and Processing in Emerging Workers, Gynes and Males of the Wasp *Polistes metricus*

**DOI:** 10.3390/insects14070617

**Published:** 2023-07-09

**Authors:** Daniel R. Gay, Timothy M. Judd

**Affiliations:** Department of Biology, Southeast Missouri State University, Cape Girardeau, MO 63701, USA

**Keywords:** *Polistes*, caste determination, males, nutrients, feeding

## Abstract

**Simple Summary:**

*Polistes metricus* colonies consist of two female castes, namely workers that have active ovaries and participate in colony care and reproductives that emerge as gynes with inactive ovaries and prepare for diapause, and males that do not overwinter. Cues during the larval stage and upon eclosion have been found to affect caste determination in *Polistes* females. The relative importance of cues experienced by larvae and those upon eclosion has not been teased out. Worker-, gyne- and male-destined pupae were housed in containers devoid of cues that affect caste determination. Thus, individuals experienced all of the normal larval cues but none experienced by adults. Adults had access to high-protein and high-carbohydrate diets. If larval cues play a larger role in caste determination in *Polistes*, the workers would be expected to prefer a high-protein diet due to their active ovaries and gynes a high-carbohydrate diet to build up lipid stores to overwinter. If the cues upon eclosion play a larger role, then all females would be predicted to have a similar diet. Males were expected to have a high-carbohydrate diet. The amounts of carbohydrates and protein consumed as well as nutrients stored in the individuals were measured. The adults showed no difference in the levels of nutrients consumed. Whole-body nutrient levels did not differ between the two female castes. Males had lower protein levels. Larval cues alone appear not to be sufficient for caste differentiation in female *Polistes*. Males appear to process the food differently than females.

**Abstract:**

There is growing evidence that paper wasps’ (*Polistes’*) fate as workers or reproductive females (gynes) is affected by cues that exist at the larval stage and during eclosion. The nutritional requirements for workers and gynes are different early in their adult lives. Males are short-lived and have different nutritional needs than females. To determine the relative importance of larval and adult cues, we reared *Polistes metricus* individuals from prepupae to adults isolated from known environmental cues shown to affect caste differentiation. Individuals were given access to two foods with different ratios of protein and carbohydrates. Levels of protein, amino acids, carbohydrates and lipids were measured after the feeding trials. If larval experience drove feeding behavior in adults, we expected to see differences in protein and carbohydrate intake as well as differences in nutrient levels. Females showed no differences in feeding or nutrient levels. Males had lower levels of protein and amino acids than females but had similar feeding results to females.

## 1. Introduction

Social Hymenoptera are well known for having distinct castes, specifically a reproductive caste (queens) and a caste that forgoes reproduction to help with brood care (workers). It has become increasingly clear that unlike social insects with distinct morphological castes in which cues during the larval stage seem to drive most of the caste differences [1,2,3,4,5,6,7], the behavior and physiology of females of eusocial wasps without morphological caste differences are affected by cues experienced as larvae and cues during the time of eclosion as adults. *Polistes* has served as a model system for primitively eusocial wasps that lack morphologically distinct castes. In temperate climates, these wasps have an annual lifecycle. A nest is initiated by a foundress that builds the initial nest and rears the first set of workers. Workers are produced earlier in the colony cycle and are individuals that are coopted into brood care [8], construct cells and defend the nest against predators. Gynes and males are produced late in the colony cycle and do not participate in brood care, nest construction or nest defense [9]. However, workers have active ovaries [10,11], and in the absence of social cues from the queen or hungry larvae, workers can become reproductive [12,13,14] but will not overwinter. Males spend their lives seeking gynes to mate with. They die prior to winter [15]. In addition to mating with males, gynes prepare to overwinter and will become foundresses in the following nesting season. Their ovaries remain undeveloped until the following spring [9]. Thus, the actual difference between the traditional worker and gyne is non-overwintering vs. overwintering strategies.

Although larval diet [16] and vibrational signals from the queen [17] have been shown to affect the physiology and nutrient levels of female larvae, photoperiod and the presence of queens and brood in a nest upon eclosion can affect the physiology [18], behavior [14] and nutritional levels of individuals [14,19,20]. What has yet to be determined is if the larval cues or eclosion cues play a more significant role in caste differences in adults and thus the divergence in nutrient levels in workers and gynes. Workers and active reproductives of primitively eusocial wasps have access to larvae which provide them with amino acid-rich saliva [21,22,23]. Prey items (caterpillars) also offer an additional nitrogen source to wasps in nests actively caring for larvae [24,25]. These resources are less available to gynes and males which appear at the tail end of the colony’s development because there are few if any larvae left and foragers primarily bring back nectar [26]. Males and gynes also have access to carbohydrates during their own foraging trips to flowers.

The state of the ovaries and the need to prepare for diapause will affect the nutritional needs of female adults. Active ovaries require the acquisition of protein, while the preparation for diapause requires energy-rich nutrients for the production of lipids [8]. Indeed, Judd [14] found that adult *Polistes metricus* that emerge in the absence of brood have high lipid and lower protein levels than adults eclosing in nests with larvae. There is evidence of some storage proteins in gynes [27], but evidence suggests that upon adults emerging from diapause in spring, levels of protein are low, and protein must be acquired by adults prior to nesting [28]. Although evidence suggests that foundresses do not purposely deprive larvae of food [16], the rate of food intake in a colony is much lower when a foundress is feeding the larvae by herself [28,29]. Additionally, the queen will produce vibrational signals that affect larval lipid stores [17]. Reproductive-destined larvae (males and females) have been found to have higher lipid stores than worker-destined larvae [28]. Nutritional differences can be caused by differences in food consumed and differences in how food is processed [30]. Even if animals consume similar levels of nutrients, they can potentially process the nutrients differently. Thus, the differences in nutrient levels in larvae could be attributed to differences in rates of food intake and differences in the processing of the food due to signals from the queen [17]. Individuals may be primed to become non-diapausing (worker) or diapausing (gyne) castes, but environmental cues could change their trajectory [14].

Most of the work on the nutritional ecology of social wasps has focused on females, while males have been relatively understudied. Males are produced by the colony at the same time as gynes [15]. Previous work has shown that male larvae have similar nutritional levels as females, suggesting that all larvae are fed the same [28]. However, as adults, males do have a different nutritional profile. Males tend to build up carbohydrate stores [28] while gynes are increasing lipid stores [11,28]. The difference in nutrient stores between males and gynes makes sense in that males do not overwinter but instead spend time searching for mating opportunities [9,15] and possibly guarding territories [31,32,33,34]. Thus, males just need quick energy stores to fuel day-to-day activities [28]. These differences may result from males having different foraging preferences than females or males processing similar food items differently than females. These two possibilities are not mutually exclusive. Both males and gynes are able to gain nutrients from returning foragers as well as forage for nectar on flowers [9]. Unlike females, males rarely participate in colony care [35,36]; thus, environmental cues such as the presence of the queen and the presence of hungry larvae that may affect female diet choice probably have little effect on males.

Here we determined if natural differences in larval experience between workers and reproductives are sufficient to drive differences in nutrient intake and processing in *Polistes.* We collected individuals from field colonies after they had spun a cap to their cell, entering the prepupal stage, and reared them in an environment devoid of any cues experienced after eclosion, including photoperiod, temperature and social cues (other larvae and adults) that have been shown to affect adult physiology and behavior [14,18,20,37,38]. Thus, all individuals experienced all of the normal larval cues prior to collection but experienced the same minimal cues during pupation and upon eclosion. Adult individuals had access to two solutions, one with a 2:1 ratio of protein to carbohydrates and the other with a 1:2 ratio of protein to carbohydrates. The amounts of each solution consumed were measured, which allowed us to estimate the consumption target [30] by calculating the total protein and carbohydrates consumed. Levels of protein, amino acids, carbohydrates and lipids present in individuals were measured in individuals once the feeding trials were over. If larval experience drives food consumption and processing in adults, then the workers’ consumption target should have a higher percentage of protein, and the consumption target of gynes should have a higher percentage of carbohydrates. In addition, worker-destined individuals would be predicted to have higher internal stores of protein and amino acids, while the gyne-destined individuals would have higher internal stores of lipids as observed in the field [14]. If cues upon eclosion play a stronger role, then the consumption targets and internal nutrient levels should not differ between worker- and gyne-destined individuals. Because males need energy for mating, they were predicted to have a consumption target that was higher in carbohydrates than protein. Because males have been shown to have high levels of carbohydrates [28], males were expected to have higher levels of carbohydrates than females in this experiment as well.

## 2. Materials and Methods

### 2.1. Wasp Collections

We collected 22 worker, 11 gyne and 8 male pupae from colonies of *Polistes metricus* located in nest boxes located in Judan Creek Conservation Area in Cape Girardeau, MO, as described by Judd (2018). Worker pupae were collected on 1 July 2015, which corresponds to when they were reared, and male and gyne pupae were collected on 2 August 2015 to ensure that no worker pupae were left in the nest. These dates were chosen based on previous work with this population [14,28]. During each collection, a single pupa was taken from each nest to avoid pseudoreplication and have minimal effect on colony productivity, especially between worker and gyne production. All individuals were collected at the prepupa or early pupal stage. Three of the workers died during the early stages of the experiment and were removed from the study.

### 2.2. Wasp Rearing and Feeding Measurements

Individual wasps were housed in 946.35 mL plastic drinking cups from QuikTrip. The cups were opaque gray, which prevented most light from entering the cup. Wasps never experienced “daytime”. We placed a small piece of an established nest in the bottom of each of the containers so the individuals would have something to sit on. Two disposable 3 mL plastic pipettes, one with each of the food types, were weighed and then placed in holes in the top of the cups that were cut in the lids. One pipette contained a 2:1 ratio by weight of glucose and egg albumin in water using 20% glucose and 10% egg albumin (high-carbohydrate diet). The other pipette contained a 1:2 ratio by weight of glucose and egg albumin in water using 10% glucose and 20% egg albumin (high-protein diet). *Polistes* gain nutrients from nectar (from flowers or foragers) and larval saliva [9]. Lipids are not a major part of the adult diet and therefore were not included. Each individual had its own container and two feeding pipettes. The pupae were placed in the cups and were monitored until they emerged. Upon emergence, two pipettes of food were added to the cup and the trial started. We also prepared a separate cup without a wasp in the same way to control for weight loss in the food due to the evaporation of water from the pipettes.

The cups were kept at room temperature; light was not regulated because the cups kept the wasps in the dark, but the lights were generally off in the room. The amount of each food that was being consumed was determined by weighing the plastic pipettes that held the food every two days. The difference in weight of the pipettes was compared to the difference in weight of pipettes placed in a control cup. Food was changed every four to six days. The individuals were allowed to feed ad libitum for twenty days before they were removed from the cup, weighed and placed in a −80 °C freezer.

### 2.3. Nutrient Measurements

Levels of soluble protein, free amino acids, carbohydrates and lipids present in the individuals were determined as described by Judd [14]. Individual wasps were homogenized in 1 mL of water in a 2.0 mL microcentrifuge tube and then centrifuged at 14× *g*. Five replicates were measured for each wasp and the average was taken.

The Bradford, anthrone and phosphovanillan assays were used to measure the levels of soluble protein, carbohydrates and lipids, respectively, as described by Judd et al. [28]. We used a ninhydrin assay to measure the levels of free amino acids based on the methods of Judd (2018). The only change to the protocol was that we added 500 µL of 1:1 chloroform–methanol to the sample before starting the phosphovanillan assay to increase the amount of aliquot available for multiple measurements (after the other three assays were completed).

### 2.4. Data Analysis

#### 2.4.1. Analysis of Food Consumption

The amount of each food type consumed by each caste was compared using a MANOVA with repeated measures (proc glm in SAS).

#### 2.4.2. Analysis of Nutrient Intake

The amount of protein and carbohydrates consumed per day was calculated for each individual. The percent protein and percent carbohydrates consumed were determined from those values. These data were arcsine transformed. We used an ANOVA with repeated measures analysis (proc glm in SAS) to determine if there was a statistical difference between the percent protein consumed by the workers, gynes and males for each day. In addition, the mean values of the protein and carbohydrates consumed by each individual were used to determine the intake targets for each caste. As some of the individuals died between days 18 and 20, data from day 20 were excluded from the analysis.

#### 2.4.3. Analysis of Nutrient Levels

A MANOVA (proc glm in SAS) followed by a Tukey honest significant difference test (THSD) was used to determine if there were any differences between individual nutrient levels per unit mass for each caste.

## 3. Results

### 3.1. Food Consumption

**Figure 1:**
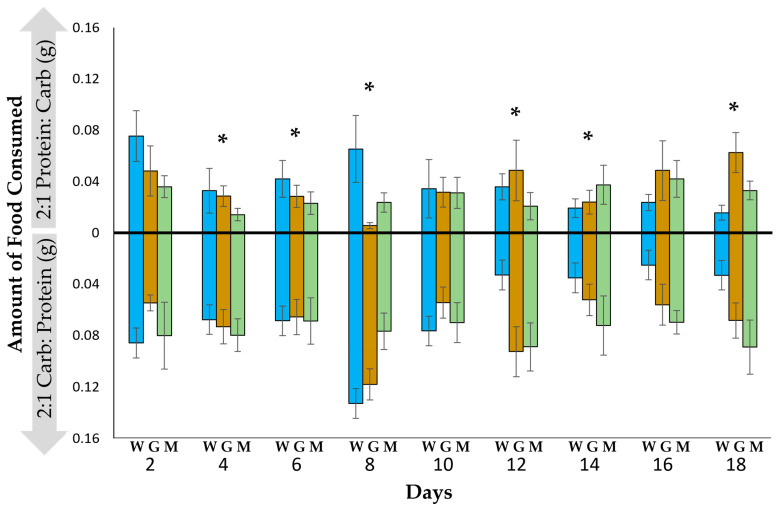
The mean (±SE) amounts of 2:1 protein–carbohydrate and 2:1 carbohydrate–protein food consumed every two days by *Polistes metricus* workers (W, N = 18), gynes (G, N = 11) and males (M, N = 8). Asterisks indicate significant differences between the amounts of food consumed (*p* < 0.05, MAVOVA repeated measures).

There was a significant difference in the amount of each food type consumed (F_(2, 34)_ = 93.01, *p* < 0.0001), but there was no effect of life stage (worker, gyne or male) (F_(2, 34)_ = 0.54, *p* = 0.71) or significant change in food intake over time (F_(2, 34)_ = 1.10, *p* = 0.42). The wasps consumed significantly more of the high-carbohydrate food than the high-protein food in 6 of the 9 days (Figure 1).

### 3.2. Comparison of Nutrient Intake

**Figure 2:**
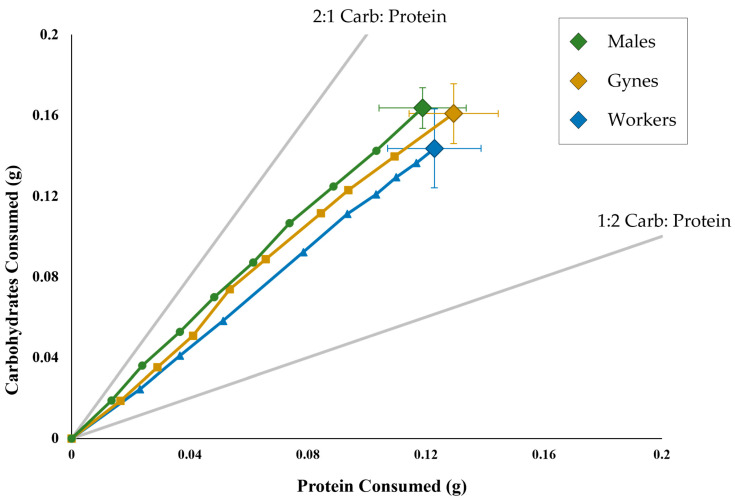
The intake of protein and carbohydrates of *Polistes* over an 18-day period. The large diamonds with error bars indicate the mean (±SE) total intake of carbohydrates and protein for worker-destined females (blue), gyne-destined females (gold) and males (green). The smaller symbols indicate the mean intake of protein and carbohydrates every two days for worker-destined females (blue triangles), gyne-destined females (gold diamonds) and males (green circles). The rails (light grey lines) indicate the amounts of protein and carbohydrates if the wasps only fed on one of the two diets (2:1 carbohydrates–protein and 1:2 carbohydrates–protein).

The overall results for the nutrient intake and total consumption showed that the males, gynes and workers were not significantly different from one another in feeding (F_(2, 35)_ = 1.49, *p* = 0.2531; Figure 2). The feeding trajectories were very similar between the three castes. There were no significant differences in daily consumption of either nutrient between the castes with the exception of days 12 and 16 (day 12: F_(2, 35)_ = 4.01, *p* = 0.0272; day 16: F_(2, 35)_ = 4.00, *p* = 0.0272). These differences might be due to a faster consumption rate by workers than males (Figure 2).

### 3.3. Comparison of Nutrient Levels

**Figure 3:**
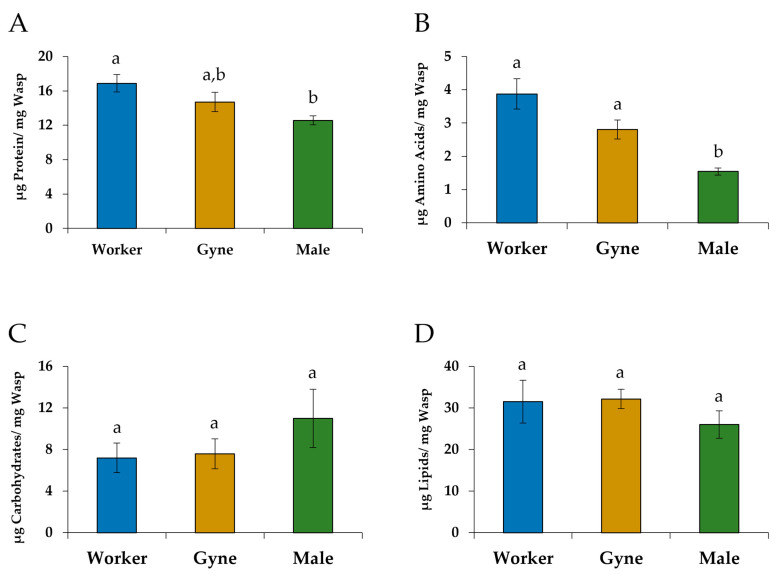
The mean (±SE) levels per unit mass of (**A**) soluble protein, (**B**) free amino acids, (**C**) carbohydrates and (**D**) lipids in worker-destined females (N = 18), gyne-destined females (N = 11) and males (N = 8) of *Polistes metricus*. Different letters indicate significant differences between castes (MANOVA and THSD).

The overall results for the nutritional assays showed that the castes were significantly different from one another (Wilks λ = 0.5210, F_(8, 62)_ = 2.99, *p* = 0.0068). Significant differences in levels of soluble protein (F_(2, 34)_ = 4.02, *p* = 0.0271) and free amino acids (F_(2, 34)_ = 10.77, *p* = 0.0002) per unit mass were found among the three groups. Males had significantly lower levels of soluble protein per unit mass than workers (*p* = 0.0233; Figure 3A). Levels of free amino acids per unit mass were significantly lower in males than in gynes (*p =* 0.0233) and workers (*p =* 0.0001; Figure 3B). There were no significant differences in the levels of carbohydrates (F_(2, 34)_ = 0.74, *p* = 0.4827; Figure 3C) or lipids (F_(2, 34)_ = 1.02, *p* = 0.3722; Figure 3D) per unit mass between the castes.

## 4. Discussion

The results of the study did not match the prediction that different castes would have different levels of protein and carbohydrate consumption. There were no significant differences in the consumption between the gynes, workers and males. All three groups preferred the high-carbohydrate diet, which suggests that they were able to distinguish between the two diets. Thus, in the absence of environmental cues upon eclosion, males and all females had the same feeding preference. These results suggest that all wasps eclosed with similar dietary predispositions, but environmental cues can shift them.

It is becoming increasingly evident that the worker phenotype of *Polistes* is expressed under certain environmental cues upon eclosion. The worker-destined or gyne-destined female wasps in this study expressed the same feeding trajectories and nutrient levels. Other studies demonstrated stark differences in behavior [14] and nutrient levels when individuals were exposed to different environmental cues [14,29]. On the other hand, studies with reduced environmental differences noted that females became more similar over time [38,39]. The fact that all females in this study ended up with similar nutrient levels despite likely having different nutrient levels as larvae [28] adds additional support to the need for environmental cues upon eclosion to reinforce the caste physiological and behavioral trajectories. The role of larval experience in caste expression may be twofold. Larvae offer amino acid-rich saliva to adults, allowing them to build protein stores [9,40]. They also induce parental care behavior in adults [8,41], causing them to forage. Foraging is energetically expensive. When provided with alternative food sources such as extra caterpillars, workers will abandon the nests [42]. Thus, when brood are present in a colony, females are trapped in a cycle with access to protein but a loss of energy while foraging and presumably never reach their intake targets. In the absence of larvae, adult females may default to the reproductive trajectory. Judd [14] found that worker-destined individuals that emerged in nests without brood had similar nutrient stores to gynes while individuals that emerged with larvae in the nest had similar nutrient levels as workers. Lipid and protein levels were distinctly different between those that had access to larvae and those that did not. Thus, adult females may default to the reproductive feeding and nutrient stores when they do not encounter the social cues from the queen or larvae upon eclosion. The fact that the worker- and gyne-destined females in this study had the same feeding behavior and nutrient stores supports this hypothesis.

Males had similar intake targets to females but had lower levels of protein and amino acids than females despite having access to the same food. Males may be absorbing less protein and amino acids than females or processing them differently [43]. Males are only produced late in the colony cycle [15] and have little interaction with larvae compared to females [35]; thus, they may not be dependent on larvae for protein as females are. Males may obtain much of the protein they need as larvae and probably do not need as much protein and amino acids for sperm production as females do for oocyte production [44]. It is possible that some of the protein was converted to muscle, which would not be detected here, but males and females naturally fly once the cuticle has hardened so they probably do not see the increase in muscle mass seen in some bees where there is a significant delay between eclosion and flight [45]. Thus, it is likely that males and females eclose with the muscle mass they need. Although not directly measured in this study, worker- and gyne-destined individuals of *P. metricus* likely eclose with different nutrient levels. Judd [28] found that worker-destined pupae had higher protein levels and lower lipid levels than gyne-destined pupae. Other studies have noted lower lipid levels in eclosing *Polistes* workers [29]. Therefore, it is likely the two female castes converged on their nutrient stores. In a natural setting, males eclose with similar nutrient levels to females but tend to increase their carbohydrate levels, presumably for energetic needs for mating and territorial defense [28]. They do not overwinter and, thus, do not need to build up lipid stores like females do. Although males accept nourishment from female foragers and may have limited contact with larvae, their nutritional target is probably not influenced by social cues.

Advanced social insects generally nest in cavities of covered nests, reducing the exposure to environmental cues during development and eclosion. Larval diet plays a larger role in female caste determination in these societies. It might be expected that a similar study with worker- and gyne-destined females from these societies would produce different results. Males are similarly ephemeral in advanced social Hymenoptera. They still gain food from workers and leave the nest on mating flights much like males in primitively eusocial wasps. Thus, it is likely that males from advanced social insects would show results similar to those seen in this study.

## Data Availability

The datasets generated and analyzed in the present study may be available from the corresponding author upon reasonable request.
